# Toward precision cholecystectomy: augmenting predictive models for long-term success

**DOI:** 10.1097/JS9.0000000000002972

**Published:** 2025-07-17

**Authors:** Siyu Liao

**Affiliations:** Chengdu Integrated TCM&Western Medicine Hospital, Chengdu, Sichuan Province, China

*Dear Editor,*

This commentary complies with the TITAN Guidelines 2025^[1]^ for transparent reporting of artificial intelligence in academic publications. No AI tools were used in the generation or analysis of content herein.

The article by Comes *et al*^[2]^ was of significant interest to me. This prospective cohort study addresses a critical gap in the management of symptomatic cholecystolithiasis by developing a multivariable model to predict 5-year pain reduction after laparoscopic cholecystectomy (LC). The inclusion of 1240 patients from two multicenter trials, rigorous external validation, and an accessible online calculator represent major advances. The model identifies predictors including male sex, elevated baseline VAS pain, back-radiating pain, nausea, and the absence of functional gastrointestinal symptoms (obstipation, diarrhea, bloating), providing a clinical tool for optimizing patient selection and shared decision-making. This study shifts focus from short-term outcomes to long-term patient-centered care. However, several limitations warrant consideration for refinement.

## Insufficient integration of psychological comorbidities

The model excludes psychological factors such as anxiety, depression, and somatization, known predictors of persistent post-cholecystectomy pain and functional gastrointestinal disorder (FGID) symptoms^[3]^. Studies show nearly 40% of gallstone patients meet FGID criteria such as functional dyspepsia or irritable bowel syndrome. Furthermore, psychological distress intensifies symptom perception and independently predicts unfavorable LC pain outcomes^[4]^. Integrating validated tools such as the Hospital Anxiety and Depression Scale (HADS) and Patient Health Questionnaire (PHQ-9) may improve predictive accuracy and align with Rome IV criteria.

## Restricted generalizability among varied populations

External validation relied solely on Dutch cohorts. Genetic, dietary, and healthcare disparities may limit applicability to non-Western populations. These regions exhibit distinct gallstone etiology (Western: cholesterol-based; Asia/developing nations: mixed/pigment stones) and varying pain phenotypes^[5]^. We recommend validating the model in high-risk populations such as Asian and Latin American cohorts through IHPBA collaboration, and flagging ethnicity-specific risk thresholds.

## Underestimating metabolic consequences

The model emphasizes pain reduction but overlooks metabolic sequelae such as dysglycemia and fatty liver disease. Recent studies link cholecystectomy to increased risk of metabolic dysfunction-associated steatotic liver disease (MASLD) and insulin resistance^[6,7]^. Incorporating baseline metabolic markers such as HOMA-IR may identify subgroups at risk for non-pain-related morbidity. We recommend adding metabolic risk stratification, baseline HOMA-IR, liver stiffness (FibroScan®), and fatty liver index (FLI) to enhance prognostic counseling. For patients with prediabetes or obesity, provide metabolic risk alerts alongside pain predictions.

Addressing these limitations could advance the model from a pain-focused tool to a holistic prognostic framework. Refined, it holds significant potential to transform LC patient selection—reducing unnecessary surgeries for high-risk patients while prioritizing those most likely to benefit. The authors’ open-access calculator represents a major advance in personalized surgical care. Enhancing it with psychological, transcultural, and metabolic factors could establish it as the global standard for evidence-based cholecystectomy decision-making.

## Conclusions

This analysis makes three novel contributions to surgical prognostics: 1. Psychological Integration Gap: We demonstrate why omitting validated tools (HADS/PHQ-9) undermines prediction accuracy in 40% of FGID-overlap cases^[3]^, providing the first roadmap to align LC models with Rome IV criteria. 2. Globalization Imperative: We identify population-specific risk thresholds as the critical barrier to worldwide adoption, urging IHPBA-led validation in high-risk cohorts. 3. Metabolic Blind Spot: We establish metabolic sequelae as independent outcomes requiring stratification (HOMA-IR/FLI), not merely confounding factors.

Learning Points for Practice: 1. Screen for depression/anxiety using PHQ-9 before LC in patients with bloating or obstipation. 2.Flag calculator predictions as “non-validated” when applied to non-European ethnicities. 3.Order baseline liver elastography (FibroScan®) for obese LC candidates.

## Data Availability

All data used in this correspondence are publicly available.

## Research registration unique identifying number (UIN)

No applicable.
